# Spinal Muscular Atrophy: The Treatment Approved

**DOI:** 10.7759/cureus.1644

**Published:** 2017-09-02

**Authors:** Rabih Tabet, Sandy El Bitar, Julie Zaidan, Garbis Dabaghian

**Affiliations:** 1 Internal Medicine, Staten Island University Hospital, Northwell Health; 2 Primary Care, Staten Island University Hospital, Northwell Health

**Keywords:** spinal muscular atrophy, neuromuscular disorders, nusinersen

## Abstract

Spinal muscular atrophy (SMA) is a rare genetic neuromuscular disorder resulting in progressive muscle weakness and atrophy. It is universally fatal, especially if the respiratory muscles are involved leading to repetitive aspiration and respiratory failure. Historically, the treatment for this disease was only supportive. Herein we describe an adult patient who presented with worsening weakness and fatigue and was subsequently diagnosed with spinal muscular atrophy. Increased awareness of this condition and a new treatment modality is required.

## Introduction

Spinal muscular atrophy (SMA) is a genetic disorder that affects the neuromuscular system causing progressive muscle weakness. SMA is universally fatal through the involvement of the respiratory muscles leading to respiratory failure. Historically, management consisted of supportive measures only. During the literature review of this case, we found that a new drug is now available in the market for the treatment of this condition. Informed consent was obtained from the patient for this study.

## Case presentation

In September 2015, a 52-year-old healthy male was seen in the outpatient department for slow progressive of dysphagia, bilateral eyelids ptosis, and generalized fatigue. History goes back to twenty years prior to his presentation when he started having trouble swallowing which slowly worsened over the years. Later on, he noticed that his upper eyelids have drooped. And around one year earlier, the patient started experiencing difficulty standing from the sitting position, and trouble climbing the stairs associated with an intermittent funny feeling in his right upper and lower extremities that he described as mild tingling and a sensation of numbness. The patient denied frank weakness but admitted to getting tired quickly. The patient had no previous medical history. He never smoked cigarettes, did not consume alcohol, and has never used illicit drugs. Upon presentation, the patient was in no distress. He was afebrile, with a blood pressure of 123/79 mmHg and a heart rate of 72 beats per minute (bpm). On physical examination, bilateral upper eyelids ptosis was noted. Otherwise, neuromuscular examination including cranial nerves examination, cerebellar function, sensation, and motor power was intact. Deep tendon reflexes in upper and lower limbs were normal. There was no muscles atrophy or tenderness, no fasciculation or twitching was noted. Laboratory tests did not show any abnormality, except for elevated creatine kinase and aldolase levels (CPK of 487 IU/L and aldolase of 8.6 U/L). The magnetic resonance imaging of the brain and whole spine was grossly insignificant. Urinary drug screening test was irrelevant and the patient denied taking any medication including herbal medicines and dietary supplements making a toxic or drug-induced etiology improbable. Thyroid stimulation hormone (TSH) level was normal ruling-out hypo- and hyperthyroid- myopathy. Anti-nuclear antibodies (ANA) and anti-neutrophil cytoplasmic antibodies (ANCA) were negative too, making autoimmune diseases less likely. Likewise, there were no autoantibodies against the acetylcholine receptors antibodies (AChR-Ab) or against the muscle-specific receptor tyrosine kinase antibody (MuSK-Ab) and an electrophysiologic study of the nerves and muscles failed to reveal any disease ruling-out myasthenia gravis. Finally, because the patient noted similar symptoms in his elder sister, an extensive genetic testing was conducted and the patient was found to have only one copy of the survival motor neuron 1 gene (SMN1) confirming the diagnosis of spinal muscular atrophy disease (SMA).

## Discussion

Spinal muscular atrophy is a rare neuromuscular disorder characterized by degeneration of the anterior horn cells in the spinal cord and motor nuclei in the lower brainstem, resulting in progressive muscle weakness and atrophy [1]. The SMA has an autosomal recessive inheritance pattern and its gene, the SMN1 gene, is found on the chromosome 5q13.2. A deficiency of the SMN1 protein does not occur unless bi-allelic deletions or mutations in this gene have taken place. Consequently, the patients who still have one intact copy of the SMN1 gene are carriers of the disease and do not manifest symptoms. However, around 2% of patients had symptomatic spinal muscular atrophy despite having one copy of the SMN1 gene. This is a case of the patient who developed symptoms despite having one intact copy of the SMN1 gene.

Currently, there are five types of SMA classified as types zero to four, depending on the age of onset and clinical course. The SMA type four is the least severe type and it occurs in adult patients with diffuse symmetric proximal muscle weakness and absent or markedly decreased deep tendon reflexes. Electromyography and muscle biopsy were once a standard part of the diagnostic evaluation for SMA, but are seldom needed nowadays for molecular genetic testing is available. Genetic testing is considered as the gold standard method to confirm the diagnosis of SMA by detection of homozygous deletions or mutations of the SMN1 gene [2].

Until last year, no treatment was available for SMA and management consisted of supportive measures directed at providing adequate nutrition, respiratory assistance, and treating or preventing complications of weakness. Currently, a new therapeutic option became available for patients with SMA. Nusinersen is the first approved drug to treat pediatric and adult patients with SMA. It has received the U.S. Food and Drug Administration approval in late December 2016 after initial clinical trials showing that it is safe, well tolerated and effective [3-5]. In particular, the unpublished ENDEAR trial showed significant improvement in motor milestones among 40% of the patients treated with Nusinersen while all untreated patients consistently deteriorated. Moreover, no significant major adverse events occurred secondary to the administration of this drug except for few reported cases of respiratory tract infections and constipation. Nusinersen is an antisense oligonucleotide that is designed to increase the expression of the survival motor neuron protein. It is administered by intrathecal injection for four initial loading doses; the first three loading doses are given at 14-day intervals, while the fourth loading dose is given 30 days after the third. Thereafter, a maintenance dose is given once every four months. Despite its cost (around $125.000/dose), treatment with Nusinersen is recommended for most patients when available since it can prevent worsening of the disease and avoid respiratory failure and death.

## Conclusions

Spinal muscular atrophy is a lethal genetic neuromuscular disorder. The patients who suffered from this disease uniformly died because of worsening muscle weakness leading to asphyxia. A new medication (Nusinersen) is readily available permitting reversal of this disease and halting the symptoms progression.
